# Quantitative proteomics analysis of tomato root cell wall proteins in response to salt stress

**DOI:** 10.3389/fpls.2022.1023388

**Published:** 2022-11-02

**Authors:** Shuisen Chen, Fei Shi, Cong Li, Quan Sun, Yanye Ruan

**Affiliations:** College of Bioscience and Biotechnology, Shenyang Agricultural University, Shenyang, China

**Keywords:** tomato, cell wall, root, salt stress, iTRAQ

## Abstract

Cell wall proteins perform diverse cellular functions in response to abiotic and biotic stresses. To elucidate the possible mechanisms of salt-stress tolerance in tomato. The 30 d seedlings of two tomato genotypes with contrasting salt tolerances were transplanted to salt stress (200 mM NaCl) for three days, and then, the cell wall proteins of seedling roots were analyzed by isobaric tags for relative and absolute quantification (iTRAQ). There were 82 and 81 cell wall proteins that changed significantly in the salt-tolerant tomato IL8-3 and the salt-sensitive tomato M82, respectively. The proteins associated with signal transduction and alterations to cell wall polysaccharides were increased in both IL8-3 and M82 cells wall in response to salt stress. In addition, many different or even opposite metabolic changes occurred between IL8-3 and M82 in response to salt stress. The salt-tolerant tomato IL8-3 experienced not only significantly decreased in Na^+^ accumulation but also an obviously enhanced in regulating redox balance and cell wall lignification in response to salt stress. Taken together, these results provide novel insight for further understanding the molecular mechanism of salt tolerance in tomato.

## 1 Introduction

Salinity is one of the most important environmental stresses affecting a wide variety of physiological and biochemical changes in crops. Salinity inhibits the growth and development of crops and disrupts metabolism, such as reducing photosynthesis, respiration and protein synthesis (Liang et al., 2018; Zörb et al., 2019). Plant roots are the primary site of salinity perception and injury, and roots sense and pass the salinity signal to the shoot for appropriate changes (Munns and Tester, 2008). The root system also plays a vital role in improving crop salt tolerance through its potential for improving access to water and limiting salt acquisition (Jung and McCouch, 2013). Therefore, the stress sensitivity of a plant’s roots limits the productivity of the entire plants. Fortunately, plants enact some mechanisms to mitigate salt stress, such as exclusion of Na^+^ from plant cells and compartmentalization of Na^+^ into vacuoles (Deinlein et al., 2014), alterations to the ultrastructure of the cell wall and subcellular organelles, and alterations to *de novo* protein biosynthesis and enzymatic activity (Ma et al., 2006).

Tomato (*Solanum lycopersicum*) is a vital vegetable with economic significance worldwide, and it has become a model species in plant research (Quinet et al., 2019). Nevertheless, most cultivated tomato species are sensitive to salt stress throughout growth and development, which restricts the production area, the quality and yield of tomato (Zaki and Yokoi, 2016; Pailles et al., 2020). The response of tomato to salt stress varies depending on the cultivar. The majority of tomato cultivars have the genetic potential of tolerance to moderate salt stress (Singh et al., 2012). To enhance the salt tolerance of tomato, the physiological responses of tomato under salt stress conditions have been extensively studied (Rivero et al., 2014; Bai et al., 2018). Transcriptomic and proteomics analyses have been performed to illuminate the responses of tomato to salt stress over the past decade (Nveawiah-Yoho et al., 2013; Gong et al., 2014; Albaladejo et al., 2018), and many genes that participate in salt tolerance have been well studied (Kou et al., 2019). However, the explicit molecular mechanisms of tomato tolerance to salt stress are still not clear.

Plant cell walls are complex and dynamic structures that are essential for the modulation of some stress signals (Komatsu and Yanagawa, 2013; Houston et al., 2016). Although cell wall proteins account for only 5~10% of the extracellular matrix mass, they perform diverse cellular functions in response to abiotic and biotic stresses (Le Gall et al., 2015; Rui and Dinneny, 2020). Among the three types of cell wall proteins, soluble proteins, weakly bound cell wall proteins and strongly bound cell wall proteins, the isolation of the strongly bound cell wall proteins was hampered by a number of technical difficulties (Jamet et al., 2006). Although the characterization of plant cell wall proteins remains challenging and requires a combination of various analytical approaches, there have been rapid advances in cell wall protein research combined with proteomics approaches (Komatsu and Yanagawa, 2013; Adelaide et al., 2018). To gain information about protein changes in cell walls, many types of stress-associated cell wall proteins have been identified in crops, and these researches have shown that cell wall proteins play an important role in stress signal transduction, cell defense and rescue, cell wall modification, etc (Wolf, 2017; Du et al., 2022; Wolf, 2022). The top leaflets showed less stress signs by salinity have an increased expression of cell wall-related genes in tomato (Hoffmann et al., 2021). Therefore, comparative proteomic analyses of the tomato cell wall could provide novel information on the underlying mechanisms of tomato responses to environmental stresses.

## 2 Materials and methods

### 2.1 Plants growth

IL8-3 (tolerant to salt stress) and M82 (sensitive to salt stress), were used in the present study. Seeds of both tomato genotypes were sterilized by a 0.2% (v/v) sodium hypochlorite solution for 10 min. Then, the seeds were rinsed extensively with deionized water. The surface sterilized seeds were germinated on moistened filter paper in the dark at 28°C for three days. The germinated seedlings were transferred onto the moistened gauze in a plastic basin (17 cm × 25 cm) for five days. The plastic basin was placed in an illuminated culture room (300 - 320 μmol m^-2^ s^-1^, 24°C day/22°C night, 16 h photoperiod). Following germination, the seedlings were grown hydroponically in a plastic container filled with Hoagland nutrient solution. Considering the nutrient requirements of tomato seedlings, the initial solution was 1/4 of the full-strength for the first 5 days, and then, the nutrient solution was replaced with 1/2 of the full-strength for another 5 days. Next, the full strength nutrient solution was used and refreshed every 5 days. When the seedlings had grown for 30 days, half of the seedlings were shifted to a nutrient solution containing 200 mM NaCl. The remaining half of the seedlings under the NaCl-free nutrient solution were used as controls. The roots and leaves were harvested on the 3^rd^ day after NaCl was added. For the cell wall proteomic analysis, the roots from each treatment were washed with distilled water and then immediately chilled in liquid nitrogen. The sample was stored at -80°C for further use. Each treatment was replicated four times.

### 2.2 Measurements of biomass, Na^+^ and K^+^ concentrations

Roots and shoots were harvested separately on the 3^rd^ day after NaCl was added. The seedlings were baked at 105°C for 15 min and then dried at 70°C to constant weight. The dry weight was weighted, and then, the seedlings (ca. 0.1000 g) were digested in concentrated HNO_3_-HClO_4_ (5:1 v/v) using a digestion block system. The Na^+^ and K^+^ concentrations were assayed using a flame photometer (FP640, Precision and Scientific Instrument, Shanghai, China).

### 2.3 Reactive oxygen species metabolism assay

The content of hydrogen peroxide (H_2_O_2_) and superoxide anion ( ${\text{O}}_{2}^{-}$ ), and the activity of superoxide dismutase (SOD) and peroxidase (POD) that from cell wall protein extraction were determined by each specific assay kit according to the the corresponding kit specification (Comin Botechnology, Suzhou, China).

### 2.4 Cell wall protein extraction

Cell wall proteins isolation was performed as previously described with modifications (Feiz et al., 2006; Francin-Allami et al., 2015). Briefly, 0.5 g (fresh weight) of roots and 0.1 g of PVPP were ground into powder using a mortar and pestle under liquid nitrogen, the powder was transferred into a 2-mL tube and filled with extracted buffer (0.6 M sucrose, 2.0 mM EDTA, 1.0 mM PMSF and 5.0 mM acetate buffer, pH 4.6). After shaking at 4°C for 30 min, the solution was centrifuged at 12,000 g for 30 min (4°C). The pellet was washed with 5 mM acetate buffer (pH 4.6). Then, the pellet was incubated with successive salt solutions as follows: twice in a 0.2 M CaCl_2_ solution (5 mM acetate buffer, 0.2 M CaCl_2_ and 10 μL protease inhibitor cocktail (Sigma-Aldrich, St. Louis, MO, USA)) for 2 h, followed by two washes in a 2 M LiCl solution (5 mM acetate buffer, 2 M LiCl and 10 μL protease inhibitor cocktail (Sigma-Aldrich, St. Louis, MO, USA)) for 2 h. Finally, CaCl_2_ and LiCl fractions were combined as cell wall fractions for further proteins precipitation. Four biological replicates were performed.

### 2.5 iTRAQ analysis

Cell wall proteins (ca. 100 μg) were reduced with 10 mM DTT for 2 h at 56°C. Then, the proteins were alkylated with 55 mM iodoacetamide at 24°C in the dark for 45 min. Then, the proteins were digested with trypsin at a 20:1 mass ratio for 12 h at 37°C. The peptide mixtures were labeled using the iTRAQ reagents 8-plex kit according to the manufacturer’s instructions (AB Sciex Inc., MA, USA). Four independent biological replicates were performed. The mixed labeled peptides were fractionated using a 4.6 × 250 mm Kindtex-C18 column (Phenomenex, Torrance, CA, USA) in a RIGOL L-3120 infinity high-performance liquid chromatography (HPLC) system (Beijing RIGOL Technology Co., Ltd., Beijing, China). LC-MS/MS analysis and mass spectrometry analysis were carried out at the National Center for Protein Science in Beijing using a TripleTOF^®^ 6600 system. ProteinPilot™ 5.0 (AB Sciex, MA, USA) software was used to analyze the raw mass spectrum data. Tandem mass spectra were extracted and searched using MS/MS data interpretation algorithms within ProteinPilot™ software 5.0 (Paragon Algorithm). The NCBI nonredundant protein database for *Solanum lycopersicum* (39020 sequences, 2020) was used for the database searching, and the mass tolerance was set to 0.05 Da. A unused confidence score of > 1.3 was used. The identified proteins with at least two matched peptides, confidence higher than 95%, and an FDR (false discovery rate)< 1% were used to perform protein quantification. Subsequently, proteins with a 2.0-fold change (p< 0.05) with good reproducibility that were detected in at least three replicates of the four biological replicates were termed differentially abundant proteins (DAPs).

### 2.6 Bioinformatics analysis

STRING (version 11.0) (https://string-db.org/) was employed to perform a protein-protein interaction analysis and statistical enrichment tests were executed for KEGG pathway annotations (Szklarczyk et al., 2015). The signal peptide sequence was predicted by SignalP (version 5.0) (Nielsen, 2017), and nonclassical secretory proteins were predicted by SecretomeP server 2.0 with an NN score above 0.6 (Bendtsen et al., 2004). The presence of functional domains and functional classification of DAPs using the *ProtAnnDB* (http://www.polebio.lrsv.ups-tlse.fr/WallProtDB/) in-house tool (San and Jamet, 2015)

### 2.7 Statistical analysis

Experimental data are presented as the means and standard deviations (SD). Each physiological parameter was examined with four biological replicates. SAS 9.2 (SAS Institute, Cary, NC, USA) with the SAS PROC ANOVA LSD model was used to perform the analysis of variance for the physiological data. A value of *p*<0.05 was considered to be statistically significant.

## 3 Results

### 3.1 Comparison of different salt-tolerant tomatoes in response to salt stress

**Figure 1:**
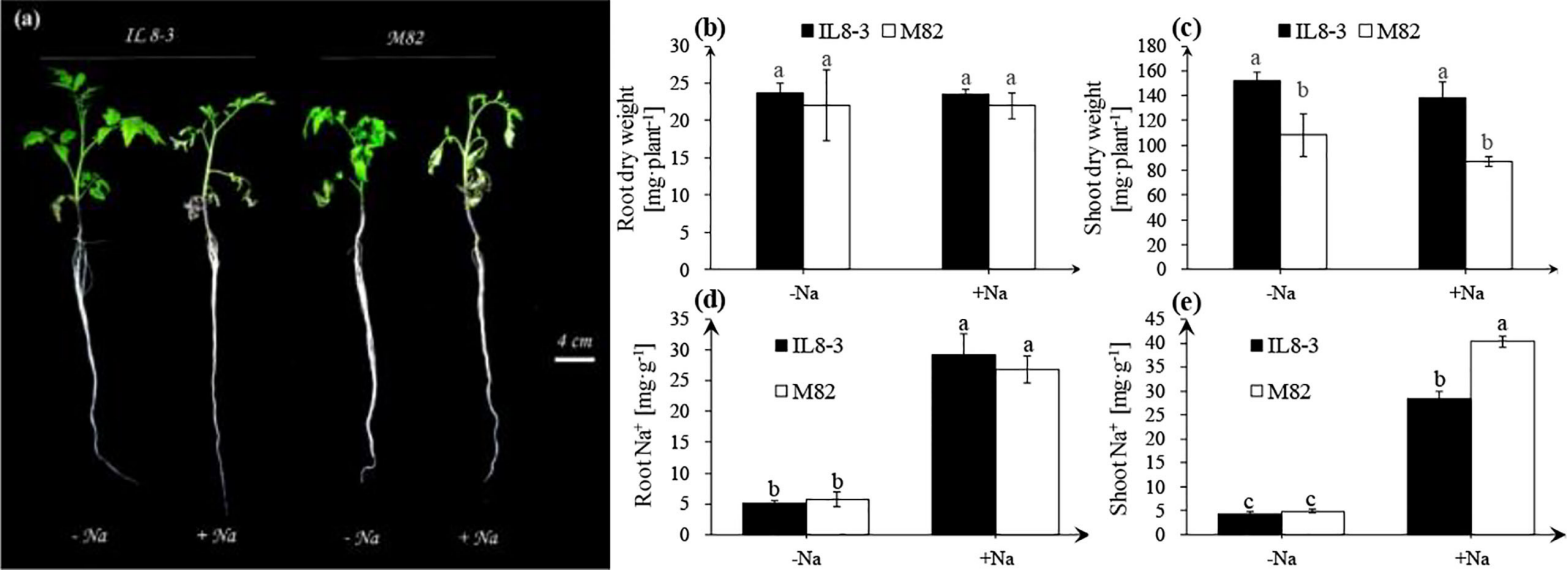
Tomato growth and physiological indices **(A)**, the alterations of root dry weight **(B)**, shoot dry weight **(C)**, root Na^+^ content **(D)**, shoot Na^+^ content **(E)** under NaCl-free condition (-Na) and 200 mM NaCl stress (+Na). The seedlings of two tomato genotypes, IL8-3 (tolerant to salt stress) and M82 (sensitive to salt stress), were grown under nutrient solution without NaCl for 30 days, then the seedlings shift to nutrient solution with or without 200 mM NaCl for 3 days. Values represent the mean ± SD of four independent replicates, bars with different letters show significant differences (ANOVA, LSD, *P*<0.05).

The physiological responses of tomato to salt stress were investigated in the present study. Compared with the NaCl-free condition, the obvious signs of dehydration in leaves were exhibited in both tomato genotypes under short-term salt stress (200 mM NaCl for 3 days), especially the salt-sensitive M82 (**Figure 1A**). Short-term salt stress did not affect the root and shoot dry weights of either tomato (**Figures 1B, C**). However, the shoot dry weight of IL8-3 (tolerant to salt stress) was significantly higher than that of M82 (sensitive to salt stress) (**Figure 1C**). Moreover, salt stress noticeably increased the Na^+^ content in the roots and shoots of both tomato genotypes (**Figures 1D, E**). The Na^+^ content of the shoots in M82 was significantly higher than that in IL8-3 under salt stress (**Figure 1E**). In addition, the salt-sensitive tomato M82 trended to accumulate more Na^+^ in shoots under salt stress, with up to 40 mg·g^-1^ Na^+^ in shoots and 27 mg·g^-1^ Na^+^ in roots. Both the Na^+^ contents in the roots and shoots of IL8-3 were 29 mg·g^-1^ Na^+^ under salt stress (**Figures 1D, E**). These results demonstrated that the two tomato genotypes with different salt tolerances had the different absorption and distribution of Na^+^ in response to short-term salt stress.

**Figure 2:**
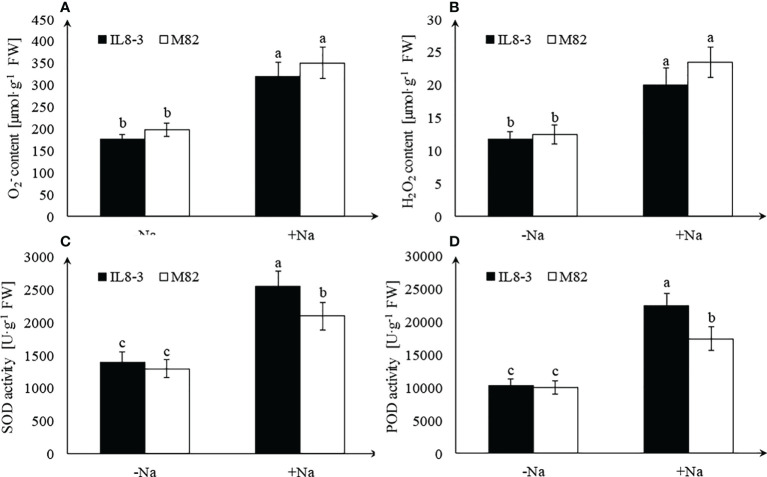
Effect of salt stress on reactive oxygen species (ROS) accumulation and antioxidant enzyme activities. ${\text{O}}_{2}^{-}$ content **(A)**, H_2_O_2_ content **(B)**, SOD activity **(C)**, POD activity **(D)**, under NaCl-free condition (-Na) and 200 mM NaCl stress (+Na). Values represent the mean ± SD of four independent replicates, bars with different letters show significant differences (ANOVA, LSD, P<0.05).

Salt stress significantly increased ${\text{O}}_{2}^{-}$ content and H_2_O_2_ content in roots of both tomato genotypes, but there was no significant difference between IL8-3 and M82 (**Figures 2A, B**). Antioxidant enzyme activities were significantly increased in roots of both tomato genotypes, and the salt-tolerant tomato, IL8-3, showed a higher SOD and POD activities under salt stress (**Figures 2C, D**).

### 3.2 Identified differential proteins of root cell wall in response to salt stress

**Figure 3:**
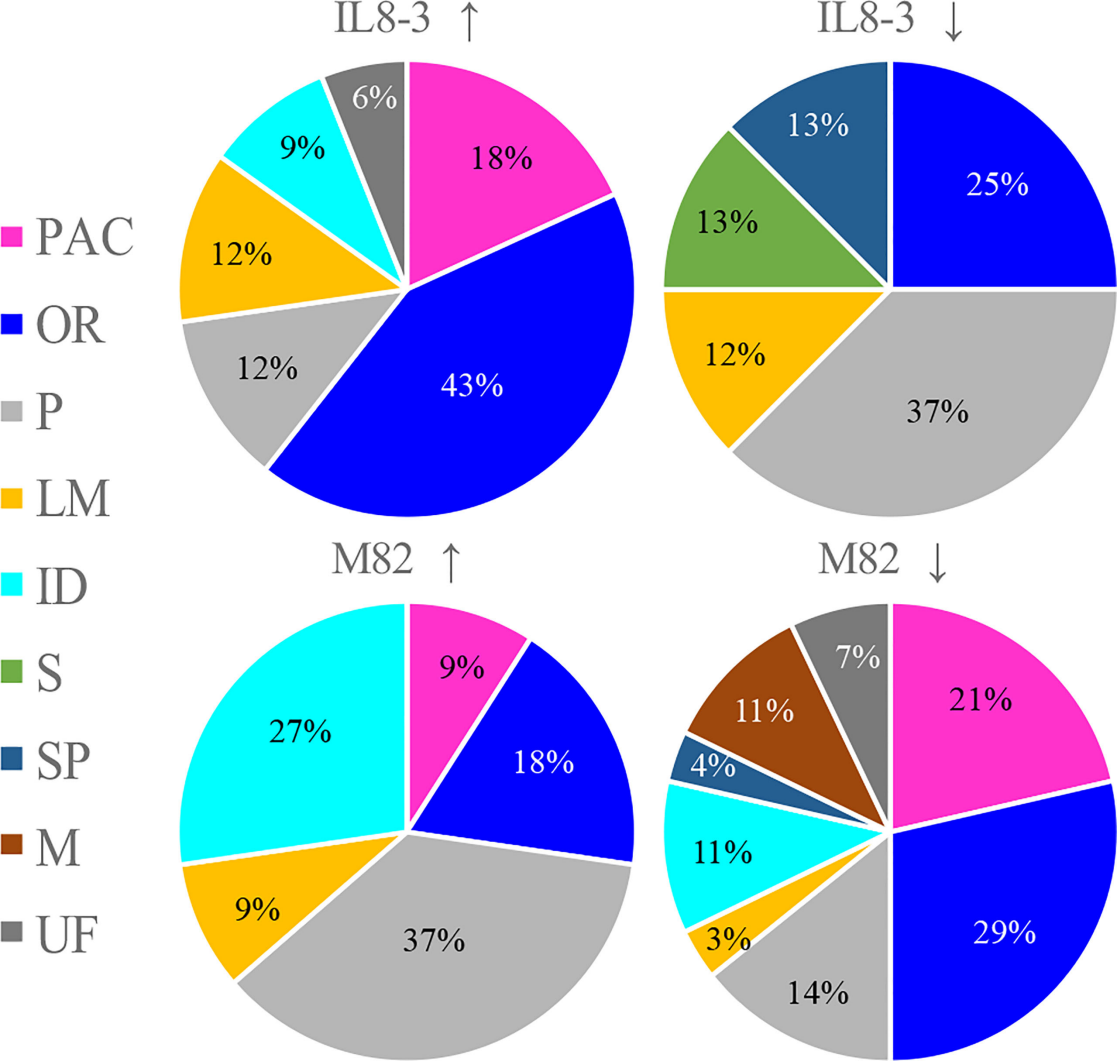
Overview showing the distribution in functional classes of the cell wall protein (http://www.polebio.lrsv.ups-tlse.fr/WallProtDB/). Cultivars with up arrow and with down arrow represent the increased and decreased in protein abundance in response to salt stress, respectively. PAC stands for proteins acting on carbohydrates, OR for oxido-reductases, P for proteases, LM for proteins related to lipid metabolism, ID for proteins with interaction domains, S for signaling, SP for structural proteins, M for miscellaneous and UF for unknown function.

To reveal the salt-tolerant mechanisms of tomato at the protein level, a comparative proteomics analysis combining isobaric tags for relative and absolute quantification (iTRAQ) and mass spectrometry was carried out on the root cell wall of the salt-tolerant tomato IL8-3 and salt-sensitive tomato M82. Finally, 82 DAPs (in the area with the white line) and 81 DAPs (in the area with the white line) in IL8-3 (**Figure S1A**) and M82 (**Figure S1B**) were selected for further analysis. There were 38 DAPs increased in protein abundance and 44 DAPs decreased in protein abundance in IL8-3 in response to salt stress. Moreover, 43 of the 82 DAPs were predicted to have signal peptides and 11 of the 82 DAPs were nonclassical secretory proteins (**Table S1**). There were 33 DAPs increased in protein abundance and 8 DAPs decreased in protein abundance of IL8-3 have the functional categories in *WallProDB* (**Figure 3**). In M82, 28 DAPs increased in protein abundance and 53 DAPs decreased in protein abundance under salt stress. Forty-five of the 81 DAPs were predicted to have signal peptides and 8 of the 81 DAPs were nonclassical secretory proteins (**Table S2**). There were 11 DAPs increased in protein abundance and 28 DAPs decreased in protein abundance of M82 have the functional categories in *WallProDB* (Figrue 3). More cell wall proteins were classified into proteins acting on carbohydrates, oxido-reductases, and proteases both in IL8-3 or M82. Cell wall proteins with interaction domains of M82 was more than that of IL8-3 (Figrue 3). In addition, 25 DAPs were identified in both IL8-3 and M82 in response to salt stress (**Table 1**). Sixteen DAPs (6 increased and 10 decreased) showed same trend in both tomato genotypes in response to salt stress (**Table 1**). Interestingly, 5 DAPs increased in IL8-3 and decreased in M82. In contrast, 4 DAPs decreased in IL8-3 and increased in M82 (**Figure S2** and **Table 1**). Based on the proteins identified, it is clear that 80% of the DAPs varied widely between IL8-3 and M82. These results indicated that IL8-3 and M82 might have different adaptive strategies in response to salt stress, at least at protein level.

**Table 1:** DAPs (differential abundant proteins) showed the same or opposite trend between salt-tolerant IL8-3 and salt-sensitive M82 under salt stress.

^※^No.	Accession #	Protein name	Fold		SP orNSP
			IL8-3	M82	
**DAPs with the same trend under salt stress**					
^CW^1	NP_001234249.1	xyloglucan-specific fungal endoglucanase inhibitor protein precursor	9.58 ± 0.02↑	4.35 ± 0.10↑	SP
^CW^2	NP_001299819.1	glucan endo-1,3-beta-glucosidase B precursor	8.05 ± 0.02↑	3.13 ± 0.11↑	SP
^CW^3	NP_001307321.1	miraculin precursor	6.37 ± 0.02↑	11.73 ± 0.03↑	SP
^CW^4	XP_004235260.1	PLAT domain-containing protein 3	3.59 ± 0.02↑	2.84 ± 0.08↑	SP
5	NP_001234099.1	alcohol dehydrogenase 2	3.54 ± 0.07↑	5.41 ± 0.03↑	-
6	NP_001234615.1	ethylene-responsive proteinase inhibitor 1 precursor	5.42 ± 0.06↑	4.13 ± 0.02↑	SP
^CW^7	XP_004245302.1	peroxidase 45-like	3.06 ± 0.96↓	16.13 ± 0.79↓	SP
^CW^8	XP_004240143.1	peroxidase 27-like	3.57 ± 0.68↓	13.72 ± 0.66↓	SP
^CW^9	XP_004247590.1	leucine-rich repeat extensin-like protein 6	3.75 ± 0.94↓	12.32 ± 1.77↓	SP
10	XP_004228473.1	60S ribosomal protein L30	3.97 ± 0.72↓	3.71 ± 0.23↓	-
11	XP_010312055.1	40S ribosomal protein S28	4.63 ± 0.84↓	20.40 ± 1.39↓	-
12	XP_010323242.1	dihydrolipoyllysine-residue acetyltransferase component 2 of pyruvate dehydrogenase complex, mitochondrial-like isoform X2	7.19 ± 0.68↓	5.30 ± 0.67↓	-
13	NP_001352840.1	peptidyl-prolyl cis-trans isomerase FKBP15-2 precursor	3.01 ± 1.43↓	6.35 ± 2.64↓	SP
14	XP_004244016.1	enhancer of mRNA-decapping protein 4-like	8.10 ± 0.96↓	3.66 ± 0.39↓	-
15	XP_004251613.1	peroxisomal fatty acid beta-oxidation multifunctional protein AIM1	3.90 ± 1.13↓	4.20 ± 1.13↓	-
16	XP_004239837.1	uncharacterized protein LOC101252396	2.86 ± 0.67↓	3.59 ± 0.38↓	SP
**DAPs with the opposite trend under salt stress**					
^CW^17	XP_004232441.1	peroxidase 72	4.26 ± 0.11↑	7.33 ± 0.48↓	SP
^CW^18	NP_001333832.1	multicopper oxidase-like protein precursor	3.27 ± 0.09↑	3.18 ± 0.33↓	SP
^CW^19	XP_004234931.1	monocopper oxidase-like protein SKS1	2.94 ± 0.06↑	3.28 ± 1.37↓	SP
^CW^20	XP_004230031.1	aspartyl protease AED3	2.68 ± 0.03↑	7.84 ± 1.24↓	SP
21	XP_004247036.1	auxin-induced in root cultures protein 12	3.97 ± 0.09↑	2.88 ± 0.73↓	SP
22	XP_004244803.1	proteasome subunit alpha type-5	2.69 ± 0.41↓	8.34 ± 0.03↑	-
23	XP_010327686.1	glutamate dehydrogenase isoform X1	3.40 ± 0.39↓	11.22 ± 0.02↑	-
24	XP_010312196.1	5-methyltetrahydropteroyltriglutamate–homocysteine methyltransferase isoform X1	2.77 ± 0.72↓	6.46 ± 0.05↑	-
25	XP_019067358.1	12S seed storage protein CRD	3.76 ± 0.83↓	3.00 ± 0.13↑	NSP

^※^ Cell wall related protein labeled CW before the No. Fold changes with up arrow ("↑") behind and with down arrow ("↓") behind represent the increased and decreased in protein abundance in response to salt stress, respectively. SP refers to the presence of a signal peptide sequence predicted by SignalP (version 4.0). NSP indicates nonclassical secretory proteins predicted by SecretomeP server 2.0 with an NN score > 0.600.

The DAPs showed opposite trend between salt-tolerant IL8-3 and salt-sensitive M82 were used to compare the difference of salt tolerance between the two tomato genotypes. There proteins only identified in IL8-3 or M82, respectively. There were 18 proteins related to cell metabolism, which 11 proteins increased in protein abundance in IL8-3 and 7 proteins decreased in protein abundance in M82 in response to salt stress (**Table 2**). For the 13 peroxidases proteins, 8 proteins increased in protein abundance in IL8-3 and 5 proteins decreased in protein abundance in M82 in response to salt stress (**Table 3**). These results indicated that the cell wall metabolism and peroxidases endowed IL8-3 with higher salt tolerance.

**Table 2:** Differentially abundant proteins (DAPs) related to cell wall metabolism that identified from IL8-3 and M82 under salt stress.

No.	Protscore	%Cov (95)	Peptide (95%)	Accession No.	Name	Fold change		
						IL8-3	M82	
1	42.89	35.0	52	XP_004232833.3	polyphenol oxidase, chloroplastic-like	6.72 ± 0.03↑	-	
2	28.45	62.1	50	XP_004250402.1	lignin-forming anionic peroxidase	5.69 ± 0.07↑	-	SP
3	146.07	53.5	565	XP_004232737.1	pectinesterase	4.01 ± 0.10↑	-	NSP
4	63.43	44.1	63	NP_001234303.1	beta-galactosidase precursor	3.52 ± 0.06↑	-	SP
5	39.35	31.3	48	XP_010324292.1	glycerophosphodiester phosphodiesterase GDPDL4	2.89 ± 0.09↑	-	SP
6	21.59	26.0	25	XP_004247400.1	pectinesterase-like	2.86 ± 0.10↑	-	NSP
7	17.84	30.6	21	NP_001234798.1	glucan endo-1,3-beta-glucosidase A precursor	2.79 ± 0.06↑	-	SP
8	46.30	42.2	39	NP_001234842.2	beta-galactosidase 4 precursor	2.46 ± 0.08↑	-	SP
9	37.39	32.3	31	XP_004245738.1	monocopper oxidase-like protein SKU5	2.70 ± 0.09↑	-	SP
10	78.72	54.5	137	XP_019070934.1	subtilisin-like protease SBT1.7	5.12 ± 0.03↑	-	SP
11	74.53	54.0	198	NP_001234774.1	subtilisin-like protease precursor	3.39 ± 0.07↑	-	SP
12	37.38	56.5	35	XP_010322133.1	alpha-galactosidase 3	-	2.86 ± 0.28↓	SP
13	20.91	27.0	18	NP_001234416.1	beta-glucosidase 08 precursor	-	4.23 ± 1.89↓	SP
14	61.04	45.0	143	NP_001233857.1	pectinesterase/pectinesterase inhibitor U1 precursor	-	7.35 ± 1.35↓	
15	14.45	56.0	19	XP_004248663.1	dirigent protein 22	-	15.19 ± 1.50↓	SP
16	35.69	31.3	26	NP_001300811.1	beta-galactosidase 5	-	3.49 ± 1.40↓	SP
17	78.39	60.4	220	XP_004232982.1	subtilisin-like protease SBT5.6	-	2.82 ± 0.76↓	SP
18	35.47	29.1	28	XP_004231026.1	subtilisin-like protease SBT1.6	-	4.15 ± 0.22↓	SP

Fold changes with up arrow (“↑”) and with down arrow (“↓”) represent the increased and decreased in protein abundance in response to salt stress, respectively. ‘-’ represent not identified. SP refers to the presence of a signal peptide sequence predicted by SignalP (version 4.0). NSP indicates nonclassical secretory proteins predicted by SecretomeP server 2.0 with an NN score > 0.600.

**Table 3:** Differentially abundant proteins (DAPs) belong to peroxidase family that identified from IL8-3 and M82 under salt stress.

No.	Protscore	%Cov(95)	Peptide(95%)	Accession No.	Name	Fold change		
						IL8-3	M82	
1	67.72	60.8	174	NP_001334412.1	peroxidase 12 precursor	15.26 ± 0.01↑	-	SP
2	93.10	82.7	464	NP_001334411.1	peroxidase 12 precursor	12.62 ± 0.00↑	-	SP
3	35.95	50.5	62	XP_004234138.1	suberization-associated anionic peroxidase 2-like	7.22 ± 0.04↑	-	SP
4	25.99	55.2	35	XP_004247506.1	peroxidase 44-like	5.38 ± 0.09↑	-	SP
5	24.78	41.1	24	NP_001296734.1	peroxidase 51 precursor	3.98 ± 0.03↑	-	SP
6	77.97	81.7	226	XP_004253400.1	peroxidase 70	3.85 ± 0.01↑	-	SP
7	34.48	50.2	46	XP_004231908.1	peroxidase 51	2.79 ± 0.06↑	-	SP
8	25.84	52.4	26	XP_004240883.1	peroxidase P7	2.79 ± 0.13↑	-	SP
9	67.98	66.0	203	NP_001334930.1	peroxidase superfamily protein precursor	-	3.55 ± 1.07↓	SP
10	49.69	74.4	110	XP_004245974.1	peroxidase 27-like	-	5.41 ± 0.62↓	SP
11	53.89	71.1	83	XP_004249055.1	cationic peroxidase 1	-	5.71 ± 1.03↓	SP
12	39.18	62.7	67	XP_004233538.1	peroxidase 72-like	-	12.94 ± 7.43↓	SP
13	44.14	74.1	96	XP_004251512.1	peroxidase 27	-	42.50 ± 2.41↓	SP

Fold changes with up arrow (“↑”) and with down arrow (“↓”) represent the increased and decreased in protein abundance in response to salt stress, respectively. ‘-’ represent not identified. SP refers to the presence of a signal peptide sequence predicted by SignalP (version 4.0). NSP indicates nonclassical secretory proteins predicted by SecretomeP server 2.0 with an NN score > 0.600.

### 3.3 Enriched pathways in which the differentially abundant proteins participated

STRING software was used to enrich the Kyoto Encyclopedia of Genes and Genomes (KEGG) pathways. In the present study, 5 pathways and 18 pathways were enriched based on the 38 DAPs that increased and 44 DAPs that decreased in IL 8-3, respectively. Fourteen pathways and 5 pathways were enriched based on the 28 DAPs that increased and 53 DAPs that decreased in M82, respectively (**Table S3**). Ten KEGG pathways were enriched in both tomato genotypes. The most significant KEGG pathways for the identified proteins were metabolic pathways (65 DAPs) and biosynthesis of secondary metabolites (43 DAPs) (**Table 4**). In addition, some of the pathways had contradictory alterations between IL8-3 and M82. The DAPs involved in the phenylpropanoid biosynthesis pathway were increased in IL8-3 but decreased in M82. While the DAPs involved in carbon metabolism, pyruvate metabolism, glycolysis/gluconeogenesis, biosynthesis of amino acids, alanine, aspartate and glutamate metabolism pathways were decreased in IL8-3, they increased in M82 (**Table 4**). These results indicated that the salt-tolerant tomato IL8-3 and salt-sensitive tomato M82 might acclimatize to salt stress through different metabolism alterations.

**Table 4:** KEGG pathway enriched based on the differentially abundant proteins (DAPs) both in IL8-3 and M82.

No.	Pathway ID	Term Description	Number of DAPs				
			IL8-3↑	IL8-3↓	M82↑	M82↓	Total
1	sly01100	Metabolic pathways	15	18	11	21	65
2	sly01110	Biosynthesis of secondary metabolites	12	10	6	15	43
3	sly00940	Phenylpropanoid biosynthesis	10	0	0	11	21
4	sly01200	Carbon metabolism	0	7	3	0	10
5	sly00620	Pyruvate metabolism	0	3	2	0	5
6	sly00010	Glycolysis/Gluconeogenesis	0	3	2	0	5
7	sly01230	Biosynthesis of amino acids	0	3	2	0	5
8	sly00250	Alanine, aspartate and glutamate metabolism	0	2	2	0	4
9	sly00190	Oxidative phosphorylation	0	3	0	3	6
10	sly00350	Tyrosine metabolism	2	0	3	0	5

The up and down arrow behind the cultivars represent increased and decreased protein abundance under salt stress, respectively.

### 3.4 Protein-protein interactions

**Figure 4:**
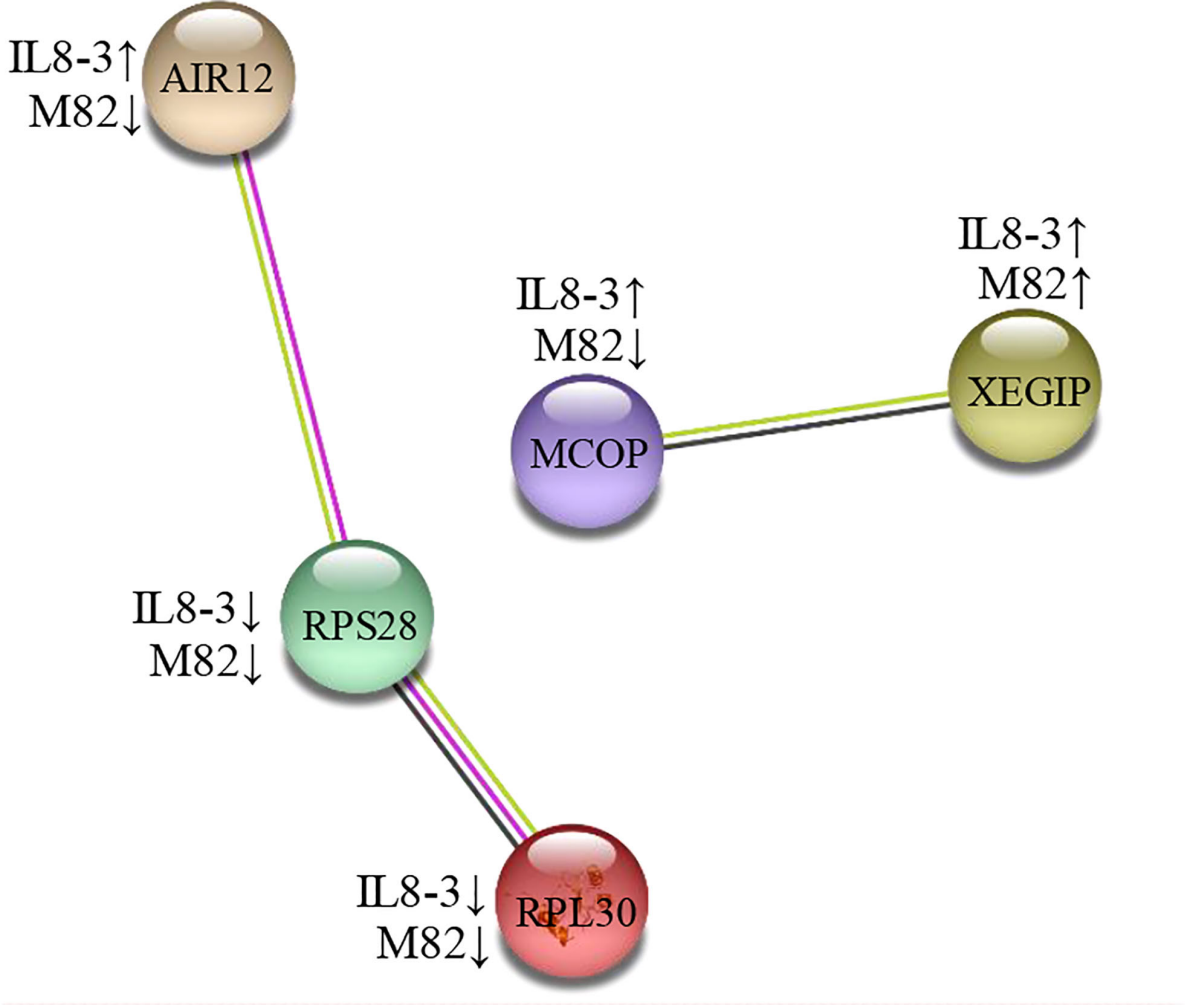
A protein interaction network of differentially abundant proteins (DAPs) under salt stress both of IL8-3 and M82. The network was built using a STRING software (https://string-db.org/) with medium confidence. The nodes represent the DAP, and the line color indicates the type of interaction evidence. The up and down arrow behind the cultivars represent increased and decreased in protein abundance under salt stress, respectively. AIR12, auxin-induced in root cultures protein 12; RPS28, 40S ribosomal protein S28; RPL30, 60S ribosomal protein L30, MCOP, multicopper oxidase-like protein precursor; XEGIP, xyloglucan-specific fungal endoglucanase inhibitor protein precursor.

To determine how tomato roots cells transmit salt signals, further analysis of the 25 DAPs identified in both tomato genotypes was performed using the STRING software with a confidence score higher than 0.5. Two groups of proteins interacting with each other were identified in the two tomato genotypes (**Figure 4**). The first group included: auxin-induced in root cultures protein 12 (AIR12), 40S ribosomal protein S28 (RPS28) and 60S ribosomal protein L30 (RPL30). AIR12 was associated with signaling, and RPS28 and RPL30 were associated with secondary metabolite biosynthesis. The second group included multicopper oxidase-like protein precursor (MCOP) and xyloglucan-specific fungal endoglucanase inhibitor protein precursor (XEGIP). MCOP was characterized as a defense-related protein, and XEGIP was characterized as a cell wall modification. In the protein interaction groups, only the AIR12 and MCOP showed the opposite changes in IL8-3 and M82 in response to salt stress.

## 4 Discussion

### 4.1 Common changes to the metabolic mechanism of IL8-3 and M82 under salt stress

Sixteen DAPs (approximately 20% of the identified DAPs) showed the same change trends under salt stress across both tomato genotypes (**Table 1**), which reflected the commonality of metabolic alterations in resistance to salt stress. Among these DAPs, ten were predicted to contain a signal peptide (**Table 1**). The DAPs that participate in cell wall modification, such as glucan endo-1,3-beta-glucosidase B precursor, which belongs to the glycoside hydrolases (GHs) family, increased in both tomato genotypes under salt stress. GHs play a key role in the degradation and reorganization of cell wall polysaccharides (Minic and Jouanin, 2006), and the alteration of cell wall polysaccharides may increase tolerance to salt stress in *Artemisia annua* (Corrêa-Ferreira et al., 2019). Xyloglucan-specific fungal endoglucanase inhibitor protein precursor (XEGIP) is involved in cell wall growth and play a vital role in plant defense. In addition, XEGIP-related proteins play a general role in protecting plants against biotic and abiotic stresses (Qin, 2003; Jones and Perez, 2014). The ethylene-responsive proteinase inhibitor 1 precursor and PLAT domain-containing protein (PLAT) 3, which are involved in signaling, also increased in both tomato genotypes under salt stress (**Table 1**). PLAT is a positive regulator of abiotic stress tolerance involved in the regulation of plant growth, and it might be a downstream target of the abscisic acid (ABA) signaling pathway (Hyun et al., 2014). In addition, the peptidyl-prolyl isomerases FKBP15-2, which negatively modulates lateral root development in *Arabidopsis* (Wang et al., 2020), also decreased in both tomato genotypes under salt stress in the present study. Taken together, these results indicated that both tomato genotypes have some common metabolic changes to resist salt stress.

### 4.2 Contrasting changes to the metabolic mechanisms of IL8-3 and M82 under salt stress

Nine DAPs (10% of the identified DAPs) showed contrasting changes between IL8-3 and M82 under salt stress. Interestingly, all 5 DAPs that predicted to have signal peptides increased in the salt-tolerant IL8-3 but decreased in the salt-sensitive M82 in response to salt stress (**Table 1**).

Cell wall localized peroxidase 72 plays an important role in lignification in *Arabidopsis* (Herrero et al., 2013). Salt stress induced the biosynthesis and deposition of lignin in the cell wall has been well reviewed (Oliveira et al., 2020). The aspartyl protease AED1 was induced locally and systemically during systemic acquired resistance signaling (Breitenbach et al., 2014). We speculated that the increase in peroxidase 72 and aspartyl protease AED3 might enhance the salt resistance of IL8-3.

Auxin-induced in root cultures protein 12 (AIR12) was interacted with RPS28 and RPL30 in the present study (**Figure 4**). AIR12 was predicted to function outside the cell, and the isolated AIRs from *Arabidopsis* were related to cell wall modification functions (Neuteboom et al., 1999). In addition, AIR12 is potentially involved in redox signaling and interacts directly with multicopper oxidase on the apoplastic side of the membrane for the directional growth of *Arabidopsis* roots (Sedbrook et al., 2002). These results indicated that in comparison to M82, the salt-tolerant tomato IL8-3 has a more positive metabolic resistance response to salt stress.

### 4.3 Differences in the mechanisms of responses to salt stress between the two tomato genotypes

Approximately 70% of the DAPs were only detected in IL8-3 or M82 respectively in response to salt stress. These results reflected that the two tomato ***genotyp*** es adapt to the salt stress by different metabolic changes.

#### 4.3.1 Cell wall modification positively regulates salt tolerance in tomato

Since the alteration of cell wall components and structures is an important adaption to saline environments, the DAPs that participate in the cell wall metabolism were compared in the two tomato genotypes with contrasting salt tolerant. Most of the proteins related to cell wall metabolism were increased in the salt-tolerant tomato IL8-3 but decreased in the salt-sensitive tomato M82, in response to salt stress (**Table 2**). These proteins resulted in alterations to cell wall polysaccharides and lignification. Pectins play a vital role in determining cell wall properties. Pectin methylesterase was positively modulates the salt tolerance of *Arabidopsis* (Yan et al., 2018). In the present study, pectinesterases was increased in IL8-3 but decreased in M82 in response to salt stress (**Table 2**). Under salt stress, the primary and secondary cell walls expanded (Le Gall et al., 2015), and the cell walls of salt-tolerant plants usually became more rigid under salt stress (Muszyńska et al., 2014). Salt stress affects the secondary cell wall formation by altering lignin biosynthesis, which increases root lignification (Oliveira et al., 2020; Kong et al., 2021). The proteins related to lignification, such as lignin-forming anionic peroxidase and monocopper oxidase-like protein SKU5 were increased in IL8-3, while the dirigent protein 22 decreased in M82 (**Table 2**). These results indicated that the higher salt tolerance of IL8-3 than that of M82 was positively related to cell wall modification.

#### 4.3.2 Peroxidases enhance the salt tolerance of tomato

Peroxidase is involved in ROS signaling and redox reactions. Plasma membrane NDPH-oxidase is activated when the plants are subjected to stress, and then, superoxide is released into the cell wall and spontaneously converted to H_2_O_2_. Peroxidase can remove H_2_O_2_ and result in the cross-linking of cell wall components (Wolf et al., 2012). In the present study, 8 peroxidases increased in IL8-3 and 5 peroxidases decreased in M82 in response to salt stress (**Table 3**). The peroxidase (POD) activity of IL8-3 was significantly higher than that of M82 in response to salt stress (**Figure 2D**). Overexpression of peroxidase also enhances the salt tolerance of soybean (Jin et al., 2019). Therefore, in comparison to M82, the salt-tolerant tomato IL8-3 has a better capacity for ROS scavenging than M82 in response to salt stress.

The peroxidase superfamily has three distantly related structural classes. Class III peroxidases containing N-terminal signal peptides secreted to the cell wall or surrounding medium and vacuoles are found in terrestrial plants (Duroux and Welinder, 2003). This class III peroxidase is mainly considered as cell wall-localized protein that plays a vital role in physiological functions and developmental processes, including cell wall hardening, pathogen penetration resistance, wounding and other abiotic stresses (Cosio and Dunand, 2009). Cell wall stiffening by peroxidases occurs mostly through lignin polymerization in cell walls (Francoz et al., 2015). Salt stress induces the gene expression of peroxidase, which has a putative role in cell wall lignification in *Ginkgo biloba* (Novo-Uzal et al., 2014). In the present study, all of the 13 peroxidases were predicted to have N-terminal signal peptides (**Table 3**). Therefore, these proteins might be different members of the class III peroxidases family, which could participate in the cell wall lignification. These results reflected that the peroxidase may facilitate tomato tolerance to salt stress and that the salt-tolerant tomato IL8-3 could better maintain the stability of the cell wall by increasing root cell wall lignification in response to salt stress.

### 4.4 Proposed molecular model of tomato salt stress

**Figure 5:**
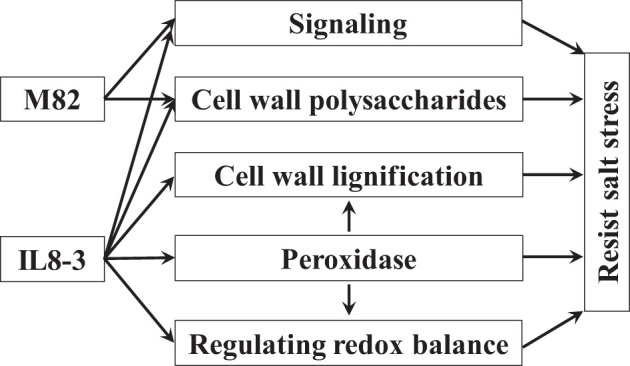
A proposed model of salt tolerance in tomato based on the cell wall proteomics of seedling roots. Arrows denote positive effects.

Based on the comparative analysis of cell wall proteomics and physiological differences between two tomato genotypes with contrasting tolerance to salt stress, a salt tolerance model of tomato was proposed (**Figure 5**). The two tomato genotypes with contrasting salt tolerances showed some common mechanisms under salt stress: the proteins involved in signaling and the cell wall polysaccharides increased in response to salt stress. In addition, the salt-tolerant tomato IL8-3 can efficiently modulate the metabolic pathways to resist salt stress. Cell wall lignification increased in IL8-3 because the proteins related to lignin metabolism increased under salt stress. Peroxidases with a signal peptide not only participates in regulating redox balance but also are involved in cell wall modification. These proteins increased under salt stress and caused IL8-3 to better regulate metabolic changes to resist salt stress.

## 5 Conclusions

Overall, a quantitative proteomic approach was performed to comprehensively study differential proteins in the cell walls of two tomato genotypes with contrasting salt tolerances. Enrichment of 82 and 81 proteins changed significantly in IL8-3 and M82, respectively. Fifty proteins were predicted to have signal peptides or nonclassical secretory proteins in both IL8-3 and M82. However, most of the proteins (70%) were only identified in IL8-3 or M82. Some common mechanisms that enable salt stress resistance, such as increasing signal transduction and altering cell wall polysaccharides, were observed in the two tomato genotypes. However, the salt-tolerant tomato IL8-3, significantly decreased Na^+^ accumulation and enhanced the regulation of the redox balance and cell wall metabolism in response to salt stress. Interestingly, these metabolic changes in the salt-sensitive M82 showed different or even opposite changes under salt stress. Compared to M82, IL8-3 better maintained plant growth, signal transduction, peroxidases activities and cell wall lignification in response to salt stress. The present study may provide novel insights for further understanding the molecular mechanisms of salt tolerance in tomato.

## Data availability statement

The original contributions presented in the study are included in the article/**Supplementary Material**. Further inquiries can be directed to the corresponding authors.

## Author contributions

SC: Methodology, Investigation, Writing-Original draft preparation. FS: Investigation, Data curation. CL: Visualization, software. QS and YR: Reviewing and Editing. All authors contributed to the article and approved the submitted version.

## Funding

This study was supported by the Science and Technology Planning Project of Shenyang Municipality (Grant No. 21-110-3-06).

## Conflict of interest

The authors declare that the research was conducted in the absence of any commercial or financial relationships that could be construed as a potential conflict of interest.

## Publisher’s note

All claims expressed in this article are solely those of the authors and do not necessarily represent those of their affiliated organizations, or those of the publisher, the editors and the reviewers. Any product that may be evaluated in this article, or claim that may be made by its manufacturer, is not guaranteed or endorsed by the publisher.
