# Supplementary material for: Quantitative proteomics analysis of tomato root cell wall proteins in response to salt stress
Source: Front Plant Sci. 2022 Nov 2;13:1023388. doi: 10.3389/fpls.2022.1023388 (PMC9666776; doi:10.3389/fpls.2022.1023388)
Supplement: Supplementary file 2 [file DataSheet_2.docx]

**Figure S1** Venn diagram of the number of differential abundant proteins (DAPs) detected in four independent biological replicates. (**a**) DAPs of salt-tolerant tomato IL8-3 and (**b**) DAPs of salt-sensitive tomato M82. Ellipse with different color represent the different biological replicates, the number within the area outlined in white was the number of DAPs for further studies.

**Figure S2.** The overlapped root cell wall differential abundant proteins (DAPs) in IL8-3 and M82 under salt stress (200 mM NaCl). Up arrow and down arrow represent increased in proteins abundance and decreased in proteins abundance of DAPs under salt stress, respectively. Each number in the overlapping area between any two circles is the number of DAPs identified in both tomato genotypes in response to salt stress.
